# Proteomic profiling of saliva reveals association of complement system with primary Sjögren's syndrome

**DOI:** 10.1002/iid3.529

**Published:** 2021-09-13

**Authors:** Mingde Li, Yajun Qi, Guizhen Wang, Su Bu, Ming Chen, Jiahui Yu, Tianyang Luo, Lulu Meng, Anran Dai, Yong Zhou, Shuai Liu, Xingxing Huo

**Affiliations:** ^1^ Experimental Center of Clinical Research, Scientific Research Department The First Affiliated Hospital of Anhui University of Chinese Medicine Hefei Anhui China; ^2^ Department of Anesthesiology, Second Clinical Medical College Anhui Medical University Hefei Anhui China; ^3^ Department of Traditional Chinese Medicine, College of Acupuncture and Massage Anhui University of traditional Chinese Medicine Hefei Anhui China; ^4^ Department of Rheumatology The First Affiliated Hospital of Anhui University of Chinese Medicine Hefei China; ^5^ Department of Traditional Chinese Pharmacology, College of Pharmacy Anhui Medical University Hefei Anhui China

**Keywords:** biomarkers, differentially expressed proteins, primary Sjögren's syndrome, salivary proteomic

## Abstract

**Introduction:**

To compare the saliva proteomes of experimental Sjögren's syndrome (ESS) model mice and healthy controls to identify potential diagnostic biomarkers for primary Sjögren's syndrome (pSS).

**Methods:**

Proteins were extracted from the saliva of three ESS and three normal control mice using the data‐independent acquisition technique. R language was used to identify the differentially expressed proteins (DEPs). Gene Ontology and Kyoto Encyclopedia of Genes and Genomes pathway analyses were performed to functionally annotate the DEPs. The protein–protein interaction (PPI) network was constructed and the core proteins were identified with the STRING website and Cytoscape software. The concentrations of Serpin family G member 1 (SERPING1), C3, complement factor H (CFH), fibrinogen alpha (FGA), and fibrinogen gamma (FGG) in saliva were determined by ELISA.

**Results:**

A total of 1722 DEPs were identified in the saliva of the ESS mice relative to the controls, of which 50 showed significantly different expression levels between the two groups. SERPING1, C3, CFH, FGA, and FGG were significantly downregulated, and keratin 4 (Krt4) and transglutaminase 3 (TGM3) were upregulated in the saliva of ESS mice. The PPI network showed that SERPING1, C3, FGG, FGA, TGM3, and hemopexin (HPX) were the core proteins. ELISA results showed that the expression of C3, CFH, FGA, and SERPING1 were significantly downregulated in the saliva of ESS mice. However, the expression of FGG was a little downregulated but with no significant difference. SERPING1, FGG, and FGA may downregulate the complement C3 by inhibiting immune complement system, thereby promoting pSS progression.

**Conclusions:**

The salivary proteome of ESS mice was markedly different from that of healthy controls, suggesting that salivary proteomics is a promising noninvasive diagnostic tool for pSS. SERPING1, C3, CFH, FGA, and FGG are potential biomarkers of pSS.

## Graphical abstract

To summarize our findings, Serpin family G member 1 (SERPING1), C3, fibrinogen alpha (FGA), fibrinogen gamma (FGG), and complement factor H (CFH) were significantly decreased in the saliva of ESS model mice and may therefore be potential diagnostic biomarkers of pSS. The role of these proteins in pSS and other autoimmune diseases (especially systemic lupus erythematosus) will have to be verified in experimental and cohort studies before possible applications in clinical diagnosis and treatment.

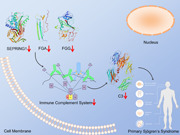

## 1 INTRODUCTION

Primary Sjögren's syndrome (pSS) is a chronic autoimmune exocrinopathy with a prevalence of approximately 0.5%. The peak incidence rate is at around 50 years of age, and it is nine times more frequent in women compared to men.^1^ At the cellular level, pSS is characterized by lymphocytic infiltration into the lachrymal and salivary glands, and B‐cell hyperactivity. The former manifests as oral and ocular sicca, fatigue, and pain, while the latter results in hypergammaglobulinemia and the appearance of serum autoantibodies.^2^ Moreover, pSS can also involve extra‐glandular sites such as the lungs, kidneys, joints, nerves, and skin.^3^ Approximately 5% of the patients develop serious complications such as B‐cell non‐Hodgkin lymphoma and diffuse large B‐cell lymphoma due to the disruption of the adaptive immune system and chronic inflammation.^3^, ^4^, ^5^ Nevertheless, the pathological basis of pSS is not completely clear, and the current research focus has shifted to salivary proteomic analysis to elucidate the complex interplay between innate and adaptive responses.^6^

Saliva is an extracellular fluid produced and secreted by the parotid, submandibular, sublingual, and several minor salivary glands located under the oral mucosa. It is a highly suitable diagnostic medium for identifying potential biomarkers since it can be collected in a safe and noninvasive manner.^6^, ^7^, ^8^ Furthermore, the saliva contains proteins, genes, antibodies, cytokines, and microbes, which are reliable indices of physiological and pathological states.^9^ For example, the presence of the complement cleavage product C3c in the saliva is an indicator of periodontitis,^10^ and microRNA in the saliva are an early diagnostic marker of oral cancer.^11^ In addition, the salivary RNA levels correlate with salivary gland inflammation in pSS patients.^12^

Several salivary protein biomarkers have been identified so far, which indicate that changes in the saliva proteome can be used to detect cancer.^13^ Furthermore, the saliva proteome has diagnostic and monitoring value for periodontal disease as well.^14^ Studies show that pSS patients exhibit distinct salivary proteomes compared to healthy individuals, with significantly elevated lactoferrin, β‐2 microglobulin, α‐enolase, and albumin.^7^, ^15^

The aim of this study was to extract and quantify the salivary proteins of experimental Sjögren's syndrome (ESS) model mice and healthy controls, to identify the core pathological proteins and potential new biomarkers of pSS.

## 2 MATERIALS AND METHODS

### 2.1 Reagents and drugs

Pentobarbital sodium, pilocarpine, and Freund's complete and incomplete adjuvants were all acquired from Sigma Chemical Co.

### 2.2 Establishment of ESS model

Specific pathogen‐free (SPF) 6‐weeks‐old female C57BL/6 mice were purchased from Vital River Laboratory Animal Technology. All mice were housed at constant temperature and humidity, and fed at libitum. All animal studies were performed in accordance with a protocol approved by the Ethics Review Committee for Animal Experimentation, Anhui University of Chinese Medicine. Bilateral salivary glands (SGs) were isolated from ten mice and homogenized at 12,000 g for 20 min at 4°C, and the supernatants were collected. BCA assay kit (Thermo Fisher Scientific) was used to determine the total protein content of the SGs. The SGs proteins were emulsified with an equal volume of Freund's complete adjuvant to a final concentration of 2.5 mg/ml. ESS was established by subcutaneously injecting the mice with 0.1 ml SGs protein emulsion in the dorsal neck and caudal base on Days 0 and 7. An unimmunized control group was included that was subcutaneously injected at the same site with 0.1 ml normal saline (also emulsified in Freund's complete adjuvant). Normal saline and SGs proteins (2.5 mg/ml) emulsified in Freund's incomplete adjuvant were injected into the respective mice on day 14 after immunization. Six weeks after immunization, the mice showing clinical symptoms of pSS were screened by salivary flow rate.

### 2.3 Salivary flow rate and body weight assessment

Salivary flow rate was measured at 6, 7, and 8 weeks after primary immunization. The mice were anesthetized by intraperitoneal injection of 2.4% pentobarbital sodium, and then injected intraperitoneally with pilocarpine (0.125 mg/kg body weight) to induce salivary secretion. Saliva was collected 5 min after injection by placing preweighed sterile dry cotton balls in the mouth for 10 min. The cotton pellets were removed and weighed, and the salivary flow rate was calculated as the total amount of saliva collected (mg/10 min). The mice were weighed at fixed time point per week after modeling, and euthanized by cervical dislocation after 8 weeks.

### 2.4 Histological assessment

The salivary gland tissues were dissected, fixed in formalin, embedded in paraffin and cut into sections. The latter were dewaxed and stained with hematoxylin and eosin as per standard protocols. The severity of lymphocytic infiltration was scored as follows: 0—no lymphocytic infiltration, 1—mild infiltration with two to eight lymphocytes, 2—mild infiltration with nine to 40 lymphocytes, 3—one lymphocytic lesion, and 4—more than two lymphocytic lesions/foci. Lymphocytic foci were defined as the presence of greater than 50 infiltrating lymphocytes infiltrates per 4 mm^2^ per tissue.^16^

### 2.5 Protein extraction and digestion

Saliva was homogenized with lysis buffer on ice for 3 min, and centrifuged at 15,000 rpm for 15 min at 4°C. The supernatants were collected and the protein concentration was determined. Fifty micrograms protein per sample was suspended for 1 h at 55°C. After alkylating in the dark for 1 h at 37°C, the samples were precipitated overnight in acetone at 20°C. The pellet was washed twice with cold acetone and then resuspended in 50 mM ammonium bicarbonate. The proteins were then digested with sequence‐level modified trypsin at 37°C for 16 h.

### 2.6 High pH reverse phase separation

The peptide mixture was redissolved and fractionated by high pH separation using Ultimate 3000 system which was performed using a linear gradient, starting from 5% B to 45% B in 40 min (B: 20 mM ammonium formate in 80% ACN). The column was re‐equilibrated at the initial condition for 15 min at a flow rate of 1 ml/min and a column temperature of 30℃. Ten fractions were collected; each fraction was dried in a vacuum concentrator for the next step.

### 2.7 Data‐dependent acquisition (DDA): Nano‐HPLC‐MS/MS analysis and database search

The peptides were redissolved and analyzed by on‐line nanospray LC‐MS/MS. A total of 3 μl peptide sample was loaded onto the analytical column, followed by a 120 min linear gradient elution, from 5% to 35% with 0.1% formic acid in ACN as solvent B. The flow rate was maintained at 200 nl/min at 40°C. The electrospray voltage of 2 kV versus the inlet of the mass spectrometer was used. The mass spectrometer was run under DDA mode, and automatically switched between MS and MS/MS mode. Raw Data of DDA were processed and statistically analyzed by Spectronaut X with default settings to generate an initial target list along with contaminant databases of mice. Trypsin was assumed as the digestion enzyme.

### 2.8 Data‐independent acquisition (DIA): Nano‐HPLC‐MS/MS analysis

The peptides were re‐dissolved and analyzed by on‐line nanospray LC‐MS/MS with the procedure as DDA. The mass spectrometer was run under DIA mode, and automatically switched between MS and MS/MS mode. The parameters was: (1) MS: scan range (*m*/*z*) = 350–1200; resolution = 120,000; AGC target = 1e6; maximum injection time = 50 ms; (2) HCD‐MS/MS: resolution = 30,000; AGC target = 1e6; collision energy = 32; Stepped CE = 5%. (3) DIA was performed with variable Isolation window, and each window overlapped 1 *m*/*z*, and the window number was 60.

### 2.9 Data analysis

Raw data of DIA were processed and analyzed by Spectronaut X (Biognosys AG) with default parameters. Retention time prediction type was set to dynamic iRT. Spectronaut Pulsar X will determine the ideal extraction window and extract data dynamically depending on iRT calibration and gradient stability. Q‐value (false discovery rate [FDR]) cutoff on precursor and protein level was applied 1%. Decoy generation was set to mutate and apply a random number of AA position swamps (min = 2, max = length/2). All selected precursors passing the filters were used for quantification. The average top 3 filtered peptides which passed the 1% Q‐value cutoff were used to calculate the major group quantities. After Student's *t* test, differently expressed proteins were filtered if their Q‐value ≤ 0.05 and Absolute AVG log_2_ ratio ≥ 0.58.

### 2.10 Identification and functional annotation of differently expressed proteins (DEPs)

The DEPs between the ESS and control groups were identified using R language. The Q‐value was calculated using student's *t* test, and corrected for multiple hypothesis testing to reduce the falsediscovery rate (FDR) obtained via the Benjamini and Hochberg (BH) method. Volcano plots and heatmaps were respectively plotted using the “ggplot2” and “pheatmap” packages in R software. The DEPs were functionally annotated by GO enrichment and KEGG pathway analyses, and the significantly enriched functions were identified by the hypergeometric test.

### 2.11 Gene set enrichment analysis (GSEA)

GSEA was performed to identify the functional DEP units involved in distinct biological processes or pathways between the ESS model group and unimmunized control group.

### 2.12 Construction of protein–protein interaction (PPI) network

The interaction network of the DEPs was analyzed using the STRING database (https://string-db.org/). The network map of proteins in the database was constructed with Cytoscape software (version 2.8.2). The proteins not included in the database were aligned with the sequences of reference proteins in the STRING database using Blastp to construct an interaction network.

### 2.13 Enzyme linked immunosorbent assay (ELISA)

The levels of C3, complement factor H (CFH), Serpin family G member 1 (SERPING1), fibrinogen alpha (FGA), and fibrinogen gamma (FGG) in saliva were quantified with an ELISA kit (BSBIO) according to the manufacturer's instructions. Briefly, the saliva samples were seeded in 96‐well plates and incubated at room temperature for 60 min. Wash it with phosphate buffered solution three times. Then the wells were firstly incubated with the primary antibody for 60 min at room temperature, and further incubated with horseradish peroxidase‐conjugated secondary antibody for 60 min at room temperature. After washing the plate three times, substrate solution was added to the plate, followed by the incubation at room temperature for 15 min. The reaction was stopped after adding a stop solution into it. Finally, optical densities were measured using a microplate reader and the absorbance value was recorded at 450 nm within less than 10 min.

### 2.14 Statistical analysis

GraphPad Prism 5.0 (GraphPad Software, Inc.) was used for all statistical analyses and graph plotting. The results were expressed as the mean ± *SD* of at least three independent experiments. Analysis of variance was used to analyze intergroup differences, and single comparisons were performed by the unpaired *t* test. A value of *p *≤ .05 was considered statistically significant.

## 3 RESULTS

### 3.1 ESS mice have a reduced salivary flow rate and extensive lymphocyte infiltration in submandibular glands

**Figure 1:**
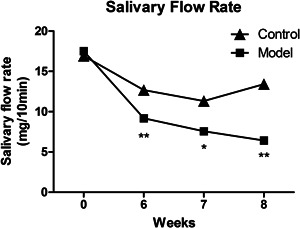
Salivary flow rate. The salivary flow rate of ESS model mice was reduced. Changes in the salivary flow rate of ESS and normal mice on weeks 6, 7, and 8. ($\overset{®}{\chi }$ ± s, *n* = 3). *p < .05, **p < .01 vs control. The $\overset{®}{\chi }$ represents the average value. ESS, experimental Sjögren's syndrome

**Figure 2:**
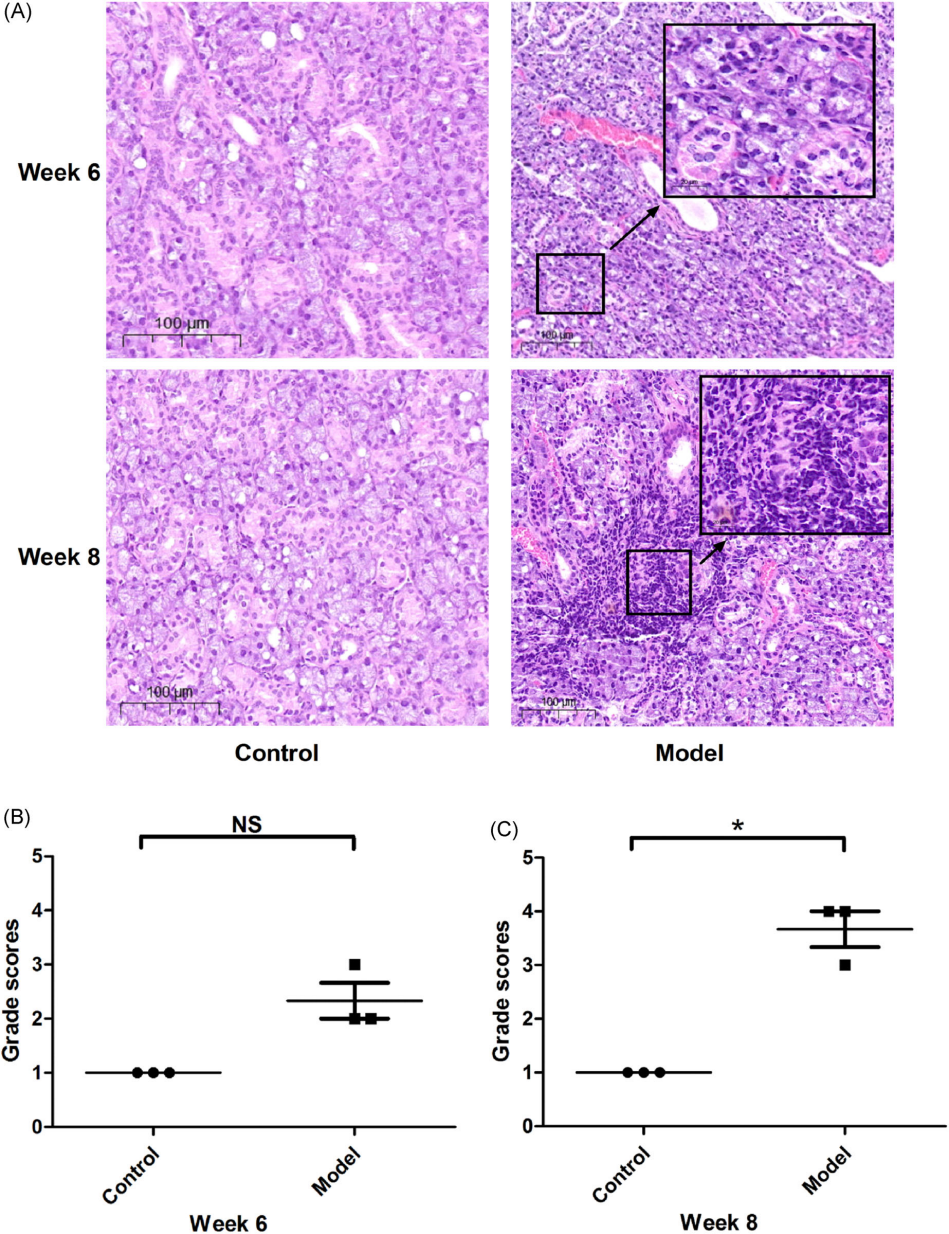
Salivary tissue and histological score. Salivary tissue (A) and histological score (B, C) of salivary tissue damage in ESS mice. Representative images showing hematoxylin and eosin‐stained salivary gland tissues harvested from the control and ESS mice after the 6th (B) and 8th (C) week. ($\overset{®}{\chi }$ ± s, *n* = 3). ^NS^ *p* > .05 versus control,*p < .05 versus control. ESS, experimental Sjögren's syndrome

Following immunization with the SG protein, the mice developed pSS‐like symptoms and exhibited a significant decrease in the salivary flow rate from the 6th week postimmunization. In contrast, the nonimmunized controls did not show any disease symptoms (Figure 1). The mean of specific values are shown in Table 1. To further confirm successful establishment of ESS, the submandibular gland tissues of the ESS model and healthy controls were analyzed for lymphocyte infiltration. Six weeks after immunization, the submandibular glands of the ESS mice showed massive lymphocytic infiltration, and multiple lymphocytic foci and massive acinar destruction were observed after 8 weeks. In contrast, no lymphocytic infiltration or tissue injury was detected in the submandibular glands of the control mice (Figure 2A). The number of infiltrating lymphocytes was quantified as previously described, and the histological score of the submandibular glands was moderately increased in mice with ESS, with no significant difference observed in the 6th week after immunization (*p* = .0572). However, there was a significant difference found compared to the histological score in the 8th week after immunization (*p* = .0153) (Figure 2B,C).

**Table 1:** Salivary flow rate

	Week 0 (mg/10 min)		Week 6 (mg/10 min)		Week 7 (mg/10 min)		Week 8 (mg/10 min)	
	Control	Model	Control	Model	Control	Model	Control	Model
Mice1	19.9	17.2	12.9	8.8	12.8	11.4	14.6	6.1
Mice2	12.3	19.6	12.5	8.5	11.8	6.1	12.3	8.2
Mice3	18.6	15.8	12.7	10.3	9.4	5.2	13.4	5.0
$\overset{®}{\chi }$	16.933	17.533	12.700	9.200	11.333	7.567	13.433	6.433
*SD*	4.065	1.922	0.200	0.964	1.747	3.350	1.150	1.626
*t* test	0.82856		0.00354		0.15931		0.00368	

Abbreviation: $\bar{\chi }$, average.

### 3.2 Identification of differentially expressed proteins

**Figure 3:**
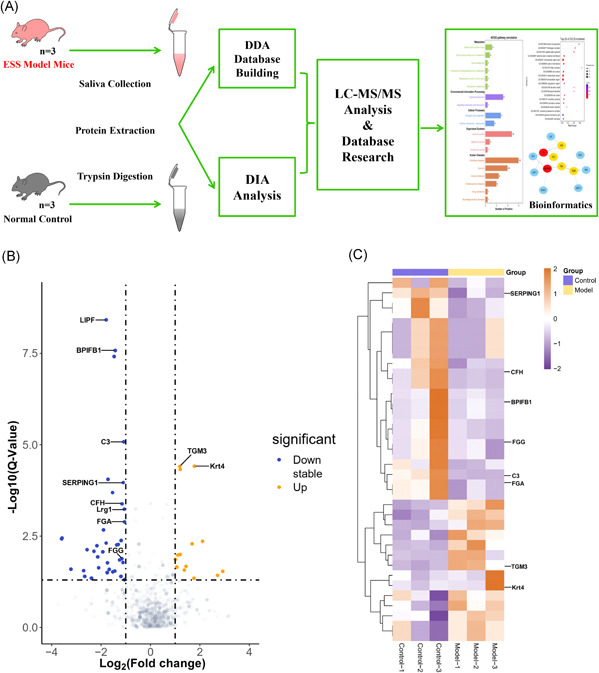
The schematic flow to study the proteome of saliva in ESS model mice (A). Volcano‐plot and heatmap of DEPs. Volcano‐plot (B) and heatmap (C) of DEPs in the saliva of ESS mice relative to the controls. Blue color indicates downregulation and orange color indicates upregulation. The x‐axis represents Q‐value and the y‐axis represents the value of log_2_ (Fold change) in volcano‐plot (B). The heatmap (C) shows 36 DEPs in the saliva of ESS mice relative to controls, including 14 upregulated (orange) and 22 downregulated (blue) proteins. DDA, data‐dependent acquisition; DEP, differentially expressed protein; DIA, data‐independent acquisition; ESS, experimental Sjögren's syndrome

The schematic flow is shown in Figure 3A. The shotgun proteomics approach identified 2261 proteins in the study based on 9084 unique peptides. Considering fold changes (log_2_ absolute) ≥ 1, Q‐value ≤ 0.05 and a maximum FDR < 1%, the significant DEPs were screened out, among which, a total of 1722 DEPs were identified in the saliva samples of ESS mice relative to the unimmunized control group, of which 50 proteins showed a significant change in their expression levels. Fourteen proteins were upregulated (keratin 4 [Krt4], transglutaminase 3 [TGM3], etc.) and 36 proteins were downregulated (LIPF, BPIFB1, C3, CFH, FGA, FGG, SERPING1, etc.) in the ESS group. The volcano map (Figure 3B) shows the distribution of the DEPs and the heatmap of the top 36 most significantly altered proteins (Figure 3C) includes 14 upregulated and 22 downregulated proteins. The top 25 differentially expressed proteins in ESS are listed in Table 2.

**Table 2:** The summary of top 25 upregulated differentially expressed proteins

Symbol	Log_2_(FC)	Q‐value	FDR	Regulated type
ADA	1.066082	2.16E−13	2.01E−10	Up
LIPF	−1.79261	3.83E−09	1.79E−06	Down
GSTO1	0.712206	2.49E−08	6.18E−06	Up
BPIFB1	−1.42749	2.65E−08	6.18E−06	Down
IGHA	−1.47181	3.87E−08	7.22E−06	Down
HPX	−0.67587	1.52E−06	0.000236	Down
LCN11	0.671456	8.07E−06	0.000969	Up
C3	−1.08325	8.32E−06	0.000969	Down
GM1553	0.996994	1.71E−05	0.001774	Up
KRT13	0.731075	3.05E−05	0.002839	Up
KRT4	1.776931	3.87E−05	0.00305	Up
IDE	1.185366	3.93E−05	0.00305	Up
TGM3	1.20637	4.73E−05	0.003388	Up
IGHG2B	−1.72157	8.84E−05	0.005888	Down
SERPING1	−1.1037	0.000108	0.006731	Down
IGKV14‐111	−1.53517	0.000203	0.011148	Down
PRB1	0.607101	0.000245	0.012707	Up
KLK10	0.762515	0.000285	0.013958	Up
CFH	−1.15312	0.000415	0.017046	Down
FUCA2	0.668382	0.000427	0.017046	Up
KLK14	0.613725	0.000439	0.017046	Up
FLNA	−0.60601	0.000572	0.020878	Down
LRG1	−1.0625	0.000582	0.020878	Down
FGG	−0.61189	0.001221	0.037938	Down
FGA	−1.05061	0.001297	0.038983	Down

Additionally, under the criterion of Q‐value ≤ 0.05 and fold changes (log_2_ absolute) ≥ 1, some preliminary proteins were not classified as DEPs, such as calmodulin (CALM) (Q‐value = 0.2897, fold changes [log_2_ absolute] = 0.9842), calmodulin‐like protein 5 (CALML5) (Q‐value = 0.2827, fold changes [log_2_ absolute] = 0.9332) and lipocalin‐2 (LCN2) (Q‐value = 0.7174, fold changes [log_2_ absolute] = 0.9153). CALM and CALML5 play a critical role in intracellular signaling and differentiation of keratinocytes, respectively. LCN2 is involved in apoptosis and the innate immune system. Aqrawi et al.^17^ have recalled the up regulated levels of LCN2, CALM, and CALML5 proteins in saliva of pSS patients, suggesting them serving as novel biomarkers, which is consistent with our experimental results.

### 3.3 GO and KEGG pathway analysis of DEPs

**Figure 4:**
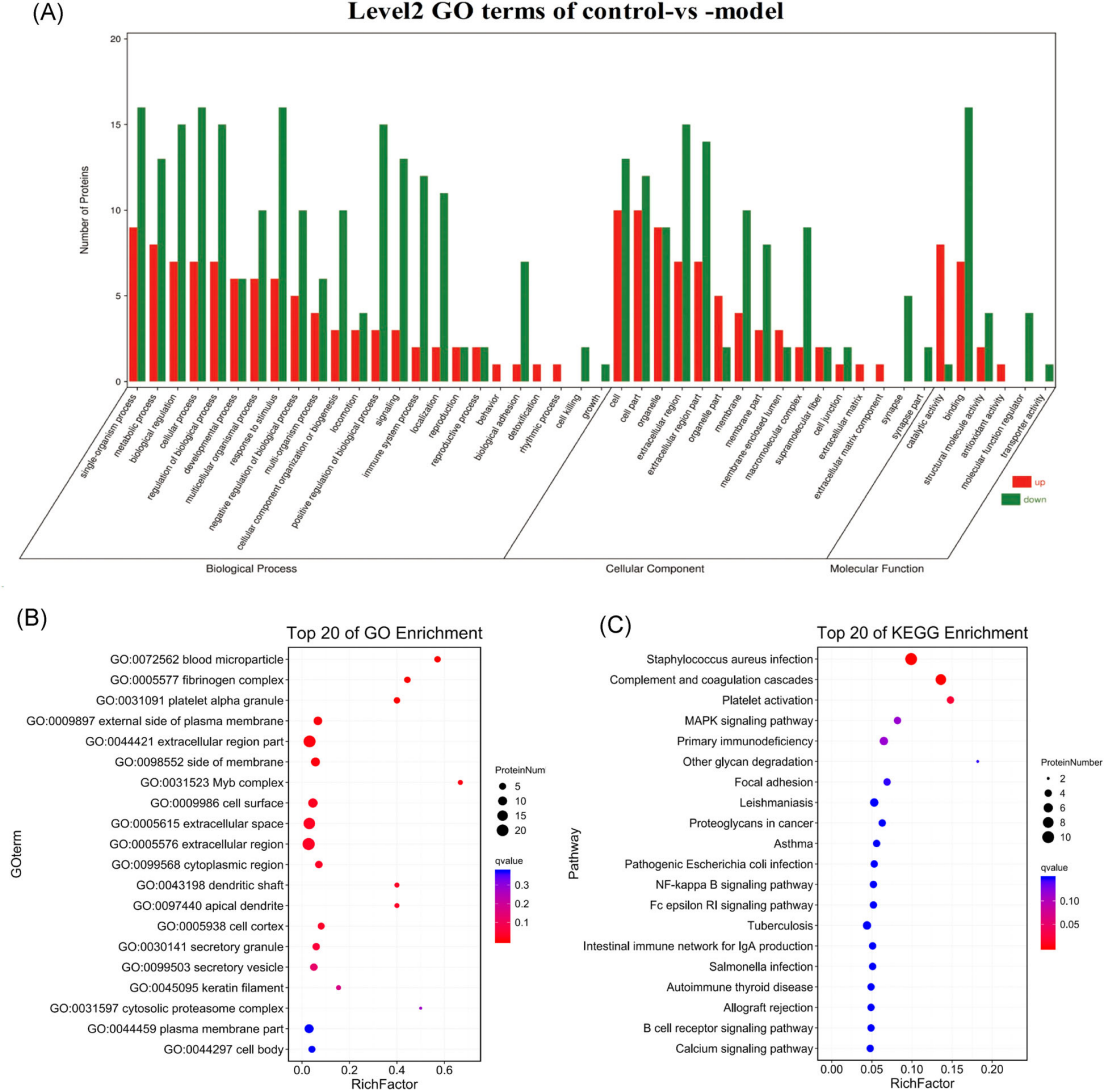
GO and KEGG enrichment bubble plot. Level2 GO terms of Control‐vs‐Model (A). The abscissa is the secondary GO term and the ordinate is the number of genes in that term. Red indicates upregulation and green indicates downregulation. GO (B) and KEGG (C) enrichment bubble plot. The top 20 GO terms and pathways with the smallest Q‐values are shown, with the ordinate as the GO term or pathway, and the abscissa as the enrichment factor. GO, Gene Ontology KEGG, Kyoto Encyclopedia of Genes and Genomes

The DEPs were functionally annotated with GO and KEGG analyses. The GO secondary classification showed that “positive regulation of biological process,” “immune system process,” “response to stimulus,” “regulation of biological process,” “cellular process,” and “biological regulation” were the most significantly enriched biological processes. In addition, “membrane part” and “macromolecular complex” were the most enriched cellular component terms, and “catalytic activity” and “binding” showed the highest enrichment among the molecular function terms (Figure 4A). Notably, 14 biological processes of two upregulated (ADA and EDE) and nine downregulated (SERPING1, C3, BPIFB1, FGA, HPX, FGG, CFH, etc.) proteins were associated with the immune system (GO: 0002376). The DEPs were mainly enriched in blood‐related biological processes, such as “blood microparticle,” “fibrinogen complex,” “platelet alpha granule,” and “Myb complex” (Figure 4B). In addition, 15 biological processes related to immune function and immune process were enriched, of which “regulation of humoral immune response,” “adaptive immune response,” “innate immune response,” “organ or tissue specific immune response,” and “mucosal immune response” were most significant (Table 3).

**Table 3:** Biological processes related to immune function and immune process

ID	Descrption	Q‐value	Num	Class
GO:0002920	Regulation of humoral immune response	0.001409	4	Biological process
GO:0002250	Adaptive immune response	0.00447	9	Biological process
GO:0045087	Innate immune response	0.005183	11	Biological process
GO:0002251	Organ or tissue specific immune response	0.005516	4	Biological process
GO:0002385	Mucosal immune response	0.005516	4	Biological process
GO:0050778	Positive regulation of immune response	0.008165	9	Biological process
GO:0002253	Activation of immune response	0.009346	8	Biological process
GO:0006959	Humoral immune response	0.009459	8	Biological process
GO:0050776	Regulation of immune response	0.022305	9	Biological process
GO:0002891	Positive regulation of immunoglobulin mediated immune response	0.026343	2	Biological process
GO:0002922	Positive regulation of humoral immune response	0.026343	2	Biological process
GO:0002684	Positive regulation of immune system process	0.029928	9	Biological process
GO:0006955	Immune response	0.034252	13	Biological Process
GO:0002824	Positive regulation of adaptive immune response based on somatic recombination of immune receptors built from immunoglobulin superfamily domains	0.034297	3	Biological process
GO:0045088	Regulation of innate immune response	0.041299	4	Biological process

**Figure 5:**
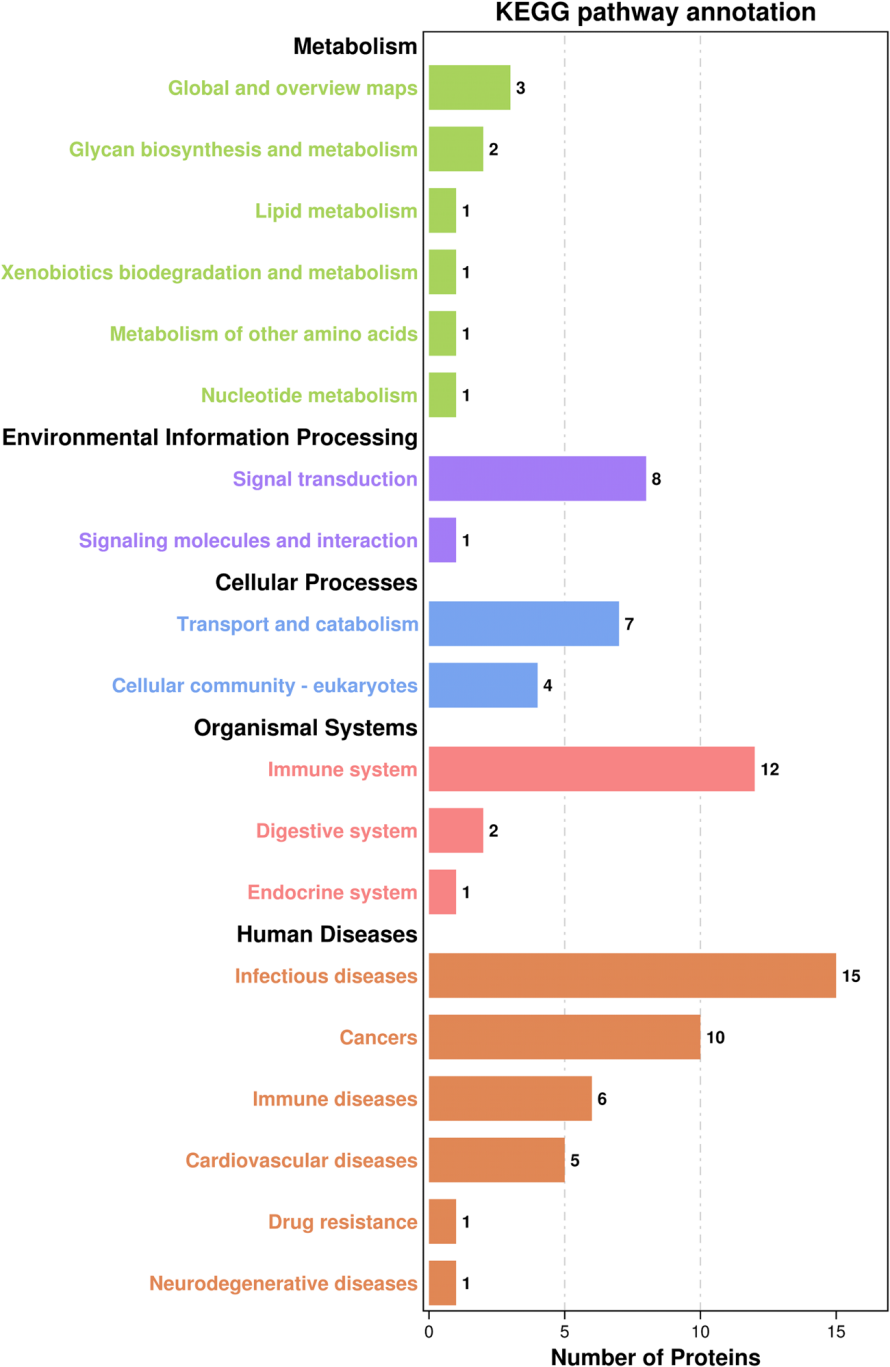
KEGG pathway annotation map. It shows 12 pathways related to the immune system, eight pathways related to signal transduction, and six pathways related to human immune system diseases. KEGG, Kyoto Encyclopedia of Genes and Genomes

The most significantly enriched KEGG pathways were “complement and coagulation cascades,” “platelet activation,” “MAPK signaling pathway,” “primary immunodeficiency,” and “other glycan degradation,” all of which are related to immune processes (Figure 4C). In addition, the DEPs were also enriched in 12 pathways associated with the immune system, eight with signal transduction and six with human immune system diseases, which are closely related to autoimmune diseases (Figure 5). C3, CFH, SERPING1, FGA, and FGG were significantly enriched in the “complex and coagulation cascades pathway,” C3 was also enriched in the “systemic lupus erythematosus” pathway, and PRB1 in the “salivary secretion” pathway (Table 4). These DEPs are potential biomarkers of pSS, and should be studied further to elucidate its underlying molecular mechanisms.

**Table 4:** KEGG pathway

KEGG (A class)	KEGG (B class)	Pathway	Q‐value	Proteins
Organismal systems	Immune system	Complement and coagulation cascades (KO04610)	0.000108144	FGG, FGA, C3, SERPING1, CFH
Organismal systems	Immune system	Platelet activation (KO04611)	0.020484053	FGG, FGA
Organismal systems	Immune system	Fc epsilon RI signaling pathway (KO04664)	0.148621916	IGHG2B, IGHA
Organismal systems	Immune system	Intestinal immune network for IgA production (KO04672)	0.148621916	IGHG2B, IGHA
Organismal systems	Immune system	B cell receptor signaling pathway (KO04662)	0.148621916	IGHG2B, IGHA
Organismal systems	Immune system	Natural killer cell mediated cytotoxicity (KO04650)	0.148621916	IGHG2B, IGHA
Organismal systems	Immune system	Hematopoietic cell lineage (KO04640)	0.178757206	IGHG2B, IGHA
Organismal systems	Immune system	Fc gamma R‐mediated phagocytosis (KO04666)	0.189438827	IGHG2B, IGHA
Organismal systems	Digestive system	Salivary secretion (ko04970)	0.425856751	PRB1
Human diseases	Immune diseases	Primary immunodeficiency (KO05340)	0.114875345	ADA, IGHG2B, IGHA
Human diseases	Immune diseases	Asthma (KO05310)	0.148621916	IGHG2B, IGHA
Human diseases	Immune diseases	Autoimmune thyroid disease (KO05320)	0.148621916	IGHG2B, IGHA
Human diseases	Immune diseases	Allograft rejection (KO05330)	0.148621916	IGHG2B, IGHA
Human diseases	Immune diseases	Systemic lupus erythematosus (KO05322)	0.228292143	C3, IGHG2B, IGHA

### 3.4 GSEA of specific pathways

**Figure 6:**
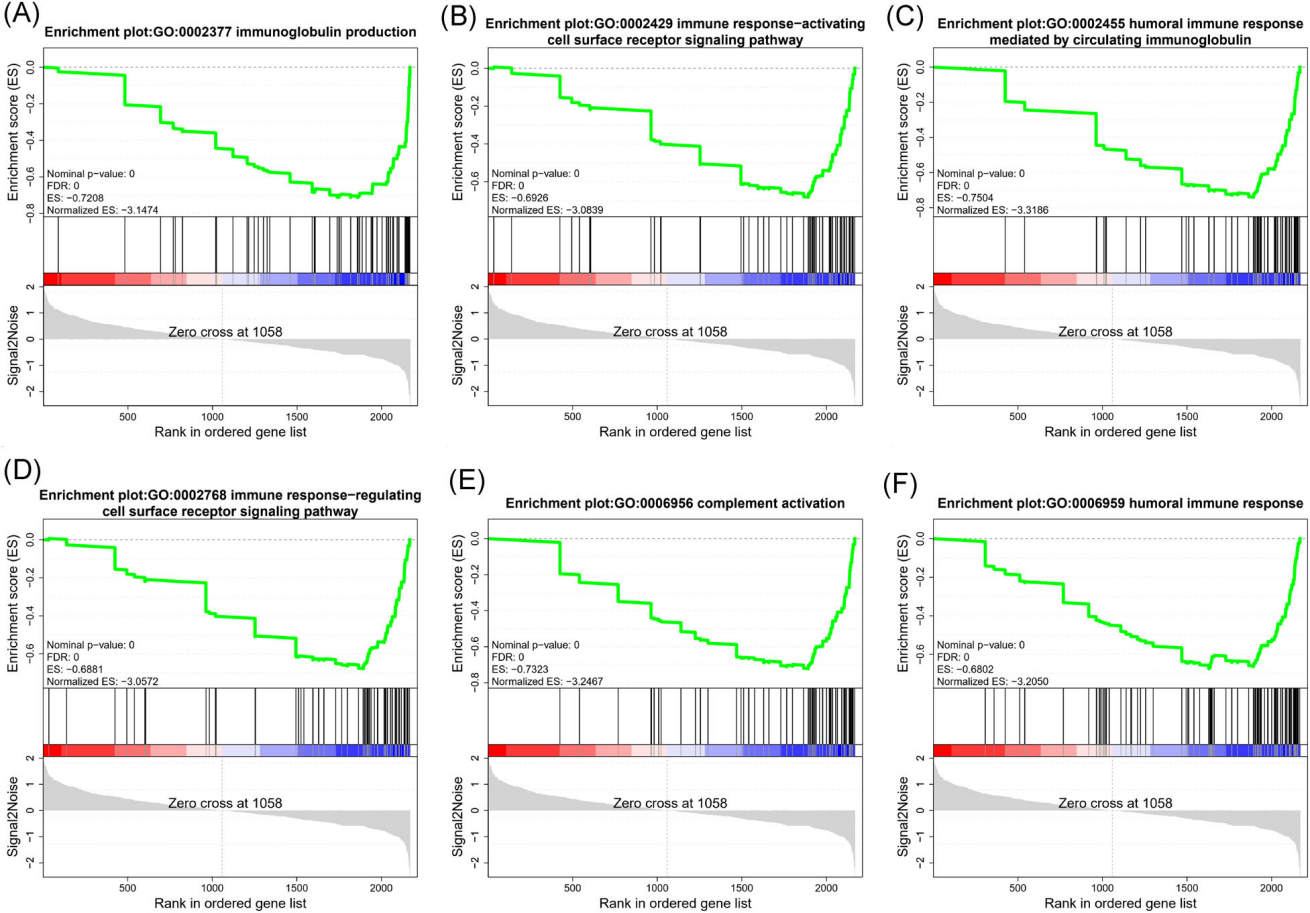
The Gene set enrichment analysis analyses of six GO terms. FDR, false‐discovery rate

**Figure 7:**
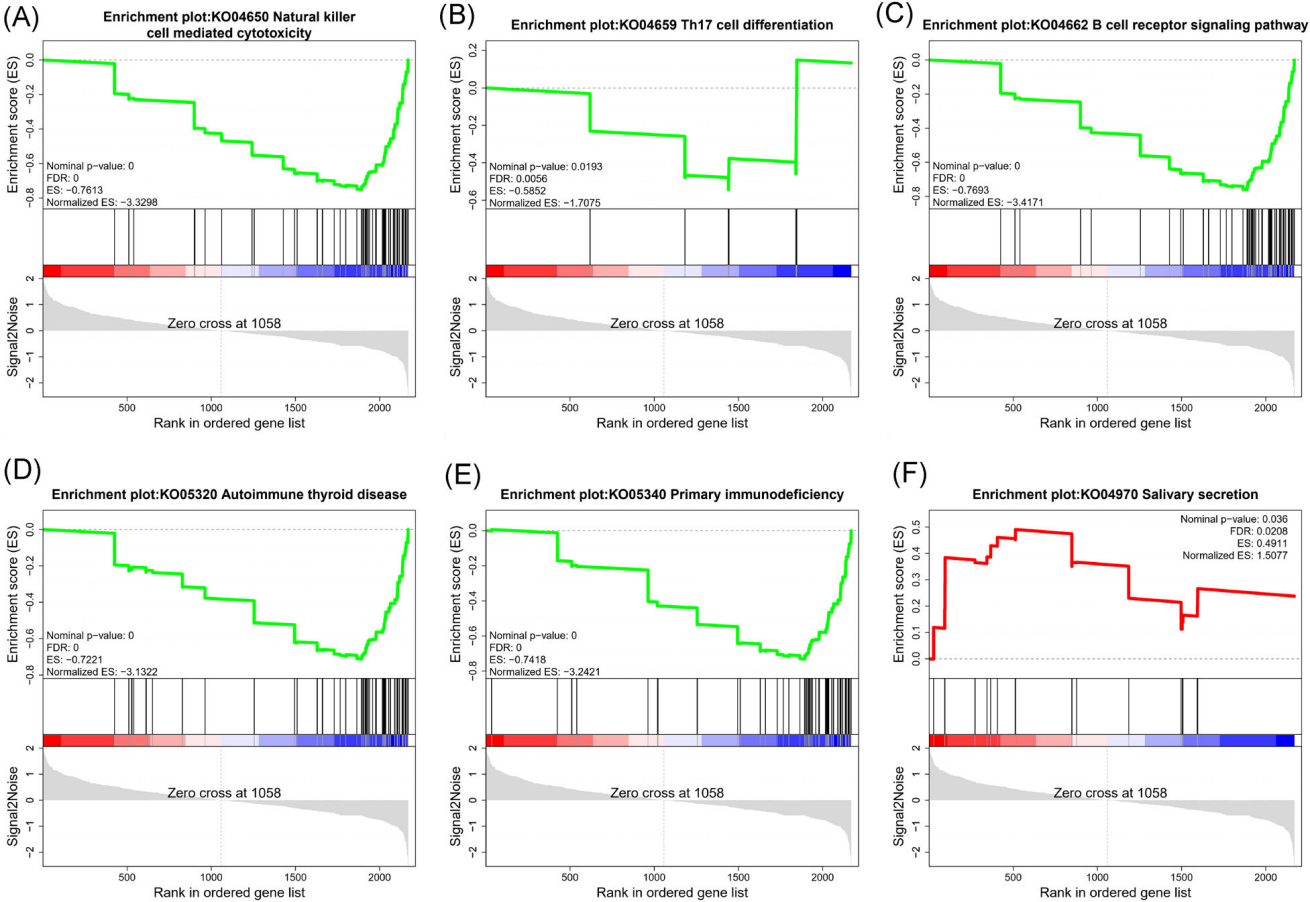
The Gene set enrichment analysis analyses of six KEGG pathways. FDR, false‐discovery rate

Most of the downregulated proteins in ESS were significantly enriched in six biological processes (nominal *p*‐value = 0) including immunoglobulin production (GO0002377), immune response‐activating cell surface receptor signaling pathway (GO0002429), humoral immune response mediated by circulating immunoglobulin (GO0002455), immune response‐regulating cell surface receptor signaling pathway (GO0002768), complement activation (GO0006956) and humoral immune response (GO0006959) (Figure 6), and five pathways including natural killer cell mediated cytotoxicity (KO04650), Th17 cell differentiation (KO04659), B cell receptor signaling pathway (KO04662), autoimmune thyroid disease (KO05320), and primary immunodeficiency (KO05340). In addition, most DEPs enriched in the salivary secretion (KO04970) pathway were upregulated in ESS (Figure 7).

### 3.5 PPI network analysis

**Figure 8:**
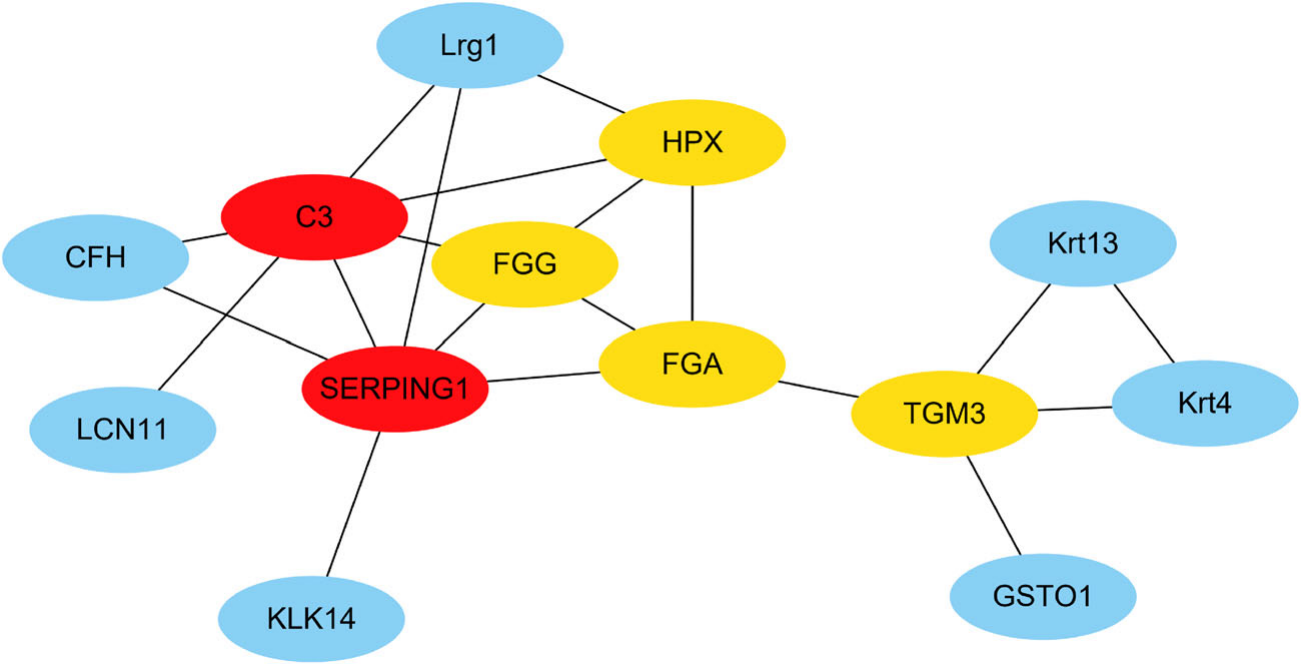
PPI network. The PPI consisting of 18 nodes and 24 edges, and the core proteins C3, SERPING1, FGG, TGM3, HPX, and FGA. FGA, fibrinogen alpha; FGG, fibrinogen gamma; HPX, hemopexin; PPI, protein–protein interaction; SERPING1, Serpin family G member 1;TGM3, transglutaminase 3

In the PPI network analysis, the DEPs, as the biomarkers of pSS, were combined and applied to Cytoscape. “Cytohubba” plugin was used to calculate the connectivity scores between the DEPs. Besides, the proteins associated with more than four nodes (degree ≥ 4) were recognized as the core proteins. Finally, the PPI network of the DEPs was consisted by 18 nodes and 24 edges, and the top 6 core proteins with the highest connectivity scores were C3, SERPING1, FGG, TGM3, HPX, and FGA, respectively, marked with red and orange ellipses. Other interacting proteins were marked with blue ellipses (Figure 8).

### 3.6 Experimental validation

**Figure 9:**
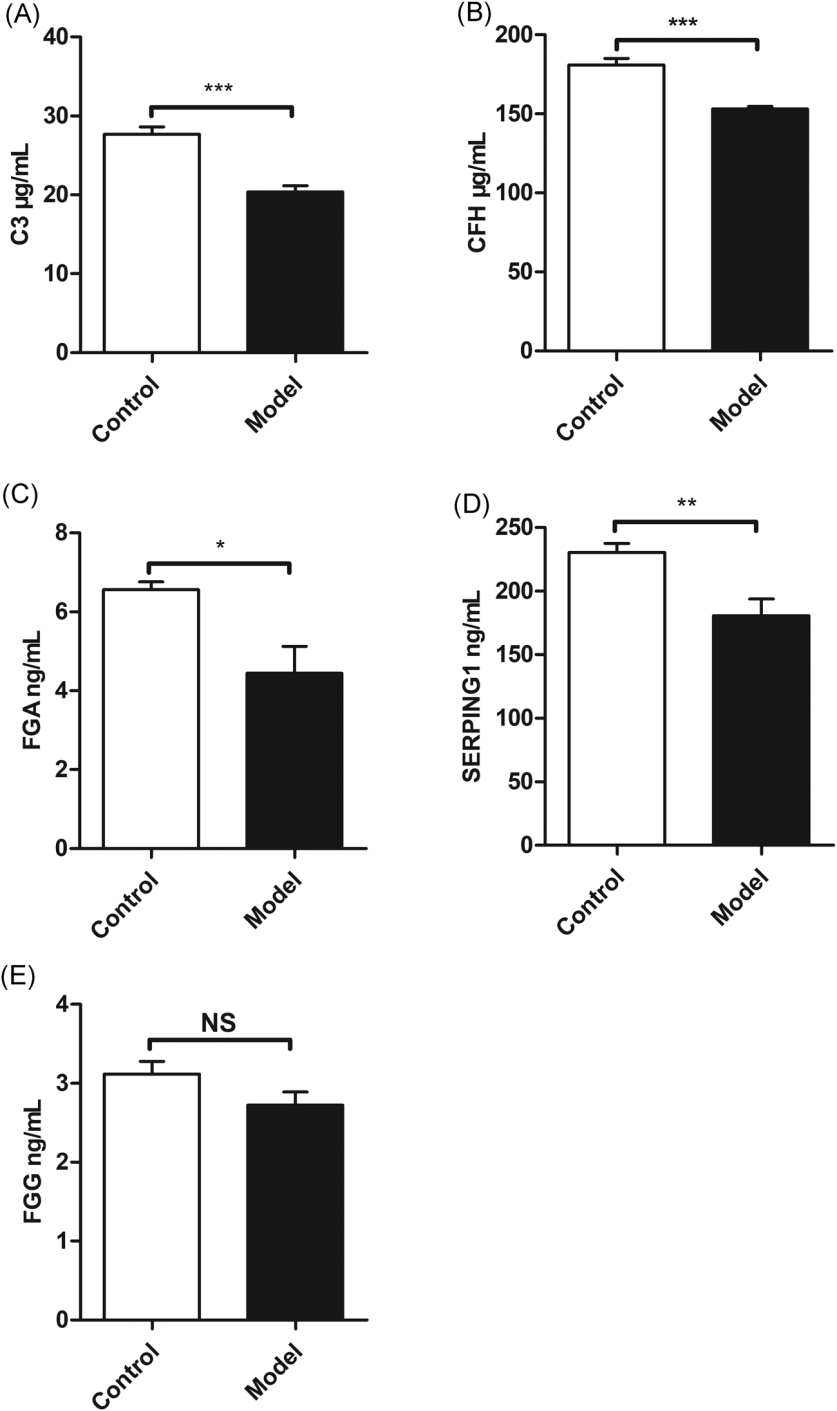
C3 (A), CFH (B), FGA (C), SERPING1 (D), and FGG (E) expression in saliva of healthy controls and ESS model mice. ESS model mice (*n*  =  3) had higher saliva levels of C3, FGG, FGA, SERPING1, CFH compared to healthy controls (*n*  =  3). ***p  <  .001, **p  <  .01, and *p  < .05, ^NS^ *p* > .05 versus the healthy controls. CFH, complement factor H; FGA, fibrinogen alpha; FGG, fibrinogen gamma; SERPING1, Serpin family G member 1

There were differences found in the expression levels of C3, CFH, SERPING1, FGA, and FGG in saliva of healthy controls and ESS model mice. As expected, compared with healthy saliva samples, C3, CFH, SERPING1, FGA, and FGG were lower expressed in the saliva samples of ESS model mice. The levels of C3 (Figure 9A), CFH (Figure 9B), SERPING1 (Figure 9D) proteins in saliva tested by ELISA were significantly lower, while the expression level of FGA (Figure 9C) protein was reduced compared with the normal group, and there was no difference found in the expression levels of FGG (*p* = 0.117) in saliva between ESS model mice and healthy controls (Figure 9E).

## 4 DISCUSSION

PSS is a relatively common autoimmune disease that affects the salivary glands and often involves multiple organs. The bioactive components in saliva are reliable diagnostic indicators of pSS. Studies show that the salivary protein expression profiles of pSS patients are markedly distinct from that of healthy subjects.^18^ High throughput analytical techniques such as proteomics and gene sequencing may provide novel insights into the pathogenesis of pSS by elucidating the global changes in the salivary proteome. The aim of this study was to compare the proteomes of the saliva from ESS model and healthy mice to identify novel biomarkers of pSS.

We successfully established the ESS mouse model by immunizing with salivary gland proteins, and observed lower salivary flow rate and submandibular lymphocyte infiltration in these mice. The proteins differentially expressed in the saliva of ESS mice relative to the controls were identified and functionally annotated through bioinformatics approaches, and the potential pSS biomarkers were screened. Fifty salivary proteins showed significantly different expression levels between the ESS and control groups, of which 14 (including Krt4 and TGM3) were upregulated and 36 (including LIPF, BPIFB1, C3, CFH, FGA, FGG, and SERPING1) were downregulated in the ESS mice. GSEA showed that most downregulated proteins are closely related to immune function, indicating that their reduced levels may affect the innate and adaptive immune responses during the progression of pSS and other autoimmune diseases, which is also in line with the current understanding of pSS pathogenesis.^3^ Furthermore, the upregulated proteins were enriched in the salivary secretion (KO04970) pathway, and may be involved in reducing saliva secretion. The downregulated proteins were enriched in the autoimmune thyroid disease (KO05320) pathway, which is consistent with the similar immunopathogenesis of pSS and autoimmune thyroid disease reported previously.^19^ These findings indicate that the altered protein expression in the saliva can affect the course of pSS, and the DEPs identified in this study may shed new light on the pathogenesis of pSS. The “cytohubba” analysis further identified C3, SERPING1, FGG, TGM3, HPX, and FGA as the core proteins. SERPING1, C3, FGA, FGG, and CFH were significantly related to autoimmune diseases, and C3 in particular is associated with systemic lupus erythematosus (SLE) that shows a similar immunopathogenic basis to pSS.^20^ ELISA results showed that C3, CFH, SERPING1, FGA, and FGG were lower expressed in the saliva of ESS mice compared with healthy control and the expression level of C3, CFH, SERPING1 and FGA exist the differences. Thus, SERPING1, C3, FGA, FGG, and CFH may serve as potential biomarkers of pSS and predict the course of the disease.

**Figure 10:**
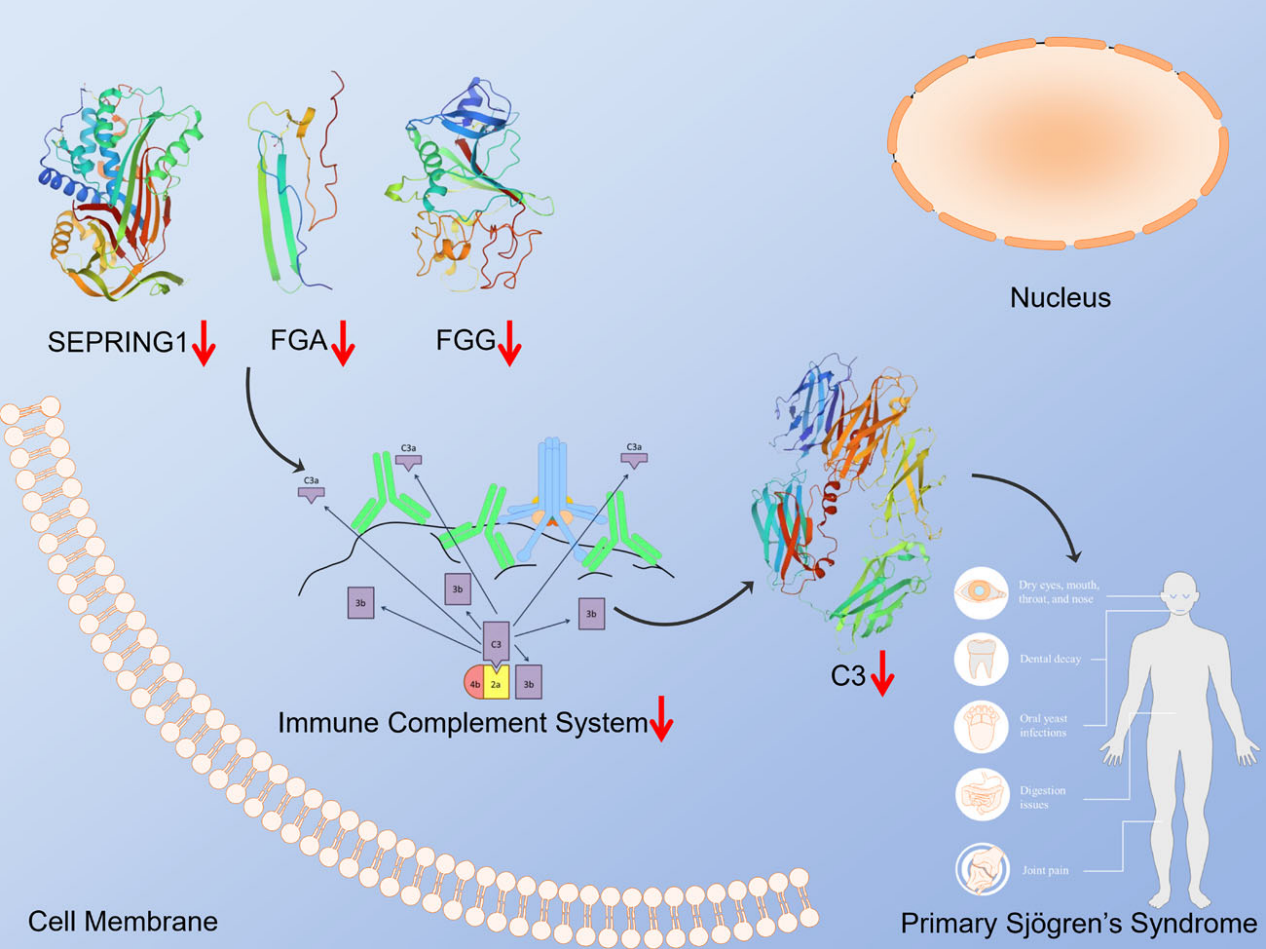
Predicted regulatory pathway. Predicted regulatory pathway of FGA, FGG, SERPING1, and C3. FGA, fibrinogen alpha; FGG, fibrinogen gamma; SERPING1, Serpin family G member 1

C3 is the most abundant complement protein in the serum, and is synthesized and secreted by macrophages and hepatic cells. Reduced level of C3 is associated with the development and exacerbation of SLE.^21^ Furthermore, Gonzalez et al. found that low expression of C3 and C4 in pSS patients correlated with increased disease activity and tissue damage.^22^ Thus, downregulation of C3 can potentially worsen the prognosis of pSS. SERPING1 is a member of SERPING family of plasmin inhibitors that degrade fibrin and other proteins, and thus regulate the coagulation pathway. Sanfilippo et al.^23^ found that SERPING1 mRNA was overexpressed in the monocytes of HIV+ patients, indicating that it likely regulates immune responses and may play a role in autoimmune diseases. CFH inhibits the alternative complement pathway and formation of C3 convertase by binding to C3b, and promotes C3b degradation as a cofactor for factor I. Lin et al. found that accompanying neuromyelitis optica spectrum disorder in the pSS patients is associated with lower CFH levels,^24^ which is consistent with the significant downregulation of CFH observed in the ESS model group in our study. We surmise that reduced CFH levels promote pSS development via overactivation of the alternative complement pathway. FGA is a member of the fibrinogen family and is synthesized by hepatocytes. It is a potential biomarker of HCC in HCV‐infected alcoholic patients.^25^ Interestingly, Wang et al. found that the elevated expression of FGA, FGG, and SERPING1 activates the immune complement system pathway,^26^ which is consistent with the decreased expression levels of all three proteins in the ESS model group. Thus, downregulation of FGA, FGG, and SERPING1 inhibits the complement system and lowers production of complement proteins like C3, resulting in autoimmune activation (Figure 10). Taken together, all these downregulated proteins, as mentioned above, are potential biomarkers of pSS and may significantly affect its occurrence and development.

Bactericidal/permeability‐increasing (BPI)‐fold‐contai‐ning family B member 1 (BPIFB1) is a member of the BPI‐fold‐containing family, which is structurally similar to BPI proteins and lipopolysaccharide binding protein (LPS). It is an innate immune receptor that can respond to external physical and chemical stimuli.^27^ Zhou et al. showed that BPIFB1 can induce proinflammatory or anti‐inflammatory cytokines in the oral cavity and upper respiratory tract, and plays a key role in triggering innate immune responses.^28^ Thus, BPIFB1 may also affect the progression of autoimmune diseases. We found that the expression level of BPIFB1 was greatly decreased in the ESS model group, suggesting that it may be responsible for the reduced salivary production in pSS patients. However, Nashida et al. detected BPIFB1 in the saliva of nonobese diabetic (NOD) mice but not in the healthy controls.^29^ The NOD model is only symptomatically similar to pSS and cannot fully mimic disease pathogenesis, whereas the ESS model induced by mouse submandibular gland proteins can simulate the pathological characteristics of pSS more accurately. Therefore, the different experimental methods and mouse strains are the likely reasons for the discrepancy in outcomes. Although our findings suggest that BPIFB1 might serve as a biomarker of pSS, its expression levels and functional relevance in pSS patients have not been demonstrated so far. The expression of Lrg1, a putative GTPase activating protein, was also decreased in the ESS model group. Chen et al.^30^ showed that Lrg1 downregulated GTPase Cdc42 and its downstream MAPKKK. Thus, downregulation of Lrg1 may promote the occurrence of pSS by activating the MAPK pathway and regulating inflammatory response. Toll‐like receptor 4 (TLR4) is a pattern recognition receptor involved in innate immune responses. Marzec et al. found that Krt4 might contribute to TLR4‐independent defense.^31^ Krt4 was upregulated in the ESS group, suggesting a possible relationship between TLRs and the progression of pSS.

Although the sample size in this study is smaller compared with previous similar studies,^17^, ^18^ the salivary protein yield and the number of identified DEPs are consistent with previous findings.^17^, ^18^, ^32^

## 5 CONCLUSIONS

To summarize our findings, C3, CFH, SERPING1, FGA and FGG were decreased in the saliva of ESS model mice and may therefore be potential diagnostic biomarkers of pSS. The role of these proteins in pSS and other autoimmune diseases (especially SLE) will have to be verified in experimental and cohort studies before possible applications in clinical diagnosis and treatment.

## CONFLICT OF INTERESTS

The authors declare that they have no conflict of interest.

## AUTHOR CONTRIBUTIONS

Xingxing Huo conceived and designed the experiments, performed the experiments, approved the final version, analyzed the data. Mingde Li and Xingxing Huo contributed to the drafting of the submitted article and the accuracy of the data analysis. Su Bu and Ming Chen contributed to the acquisition of reagents, materials and analysis tools. Lulu Meng contributed to the critical revision of the manuscript for important intellectual property. Anran Dai, Yong Zhou, and Shuai Liu contributed to the analysis and interpretation of the data. Yajun Qi, Guizhen Wang, Tianyang Luo, and Jiahui Yu contributed to perform the validation experiment and revise the manuscript.

## Data Availability

The data that support the findings of this study are available from the corresponding author upon reasonable request.
